# Metabolic Profiling of Diabetic Cats in Remission

**DOI:** 10.3389/fvets.2020.00218

**Published:** 2020-05-15

**Authors:** Susan Gottlieb, Jacquie Rand, Stephen T. Anderson, John Murray Morton, Daniel A. Dias, Berin A. Boughton, Ute Roessner, Ziad Ramadan

**Affiliations:** ^1^The Cat Clinic, Brisbane, QLD, Australia; ^2^School of Veterinary Science, The University of Queensland, Gatton, QLD, Australia; ^3^Australian Pet Welfare Foundation, Kenmore, QLD, Australia; ^4^School of Biomedical Sciences, The University of Queensland, St. Lucia, QLD, Australia; ^5^Jemora Pty Ltd, Geelong, VIC, Australia; ^6^School of Health and Biomedical Sciences, Discipline of Laboratory Medicine, RMIT University, Bundoora, VIC, Australia; ^7^Metabolomics Australia, School of BioSciences, The University of Melbourne, Melbourne, VIC, Australia; ^8^Nestlé Purina Research, St. Louis, MO, United States

**Keywords:** diabetes mellitus, metabolomics, diabetic remission, feline endocrinology, feline diabetes

## Abstract

**Background:** The majority of diabetic cats in remission have abnormal glucose tolerance, and approximately one third relapse within 1 year. Greater understanding of the metabolic characteristics of diabetic cats in remission, and predictors of relapse is required to effectively monitor and manage these cats.

**Objectives:** To identify and compare differences in plasma metabolites between diabetic cats in remission and healthy control cats using a metabolomics approach. Secondly, to assess whether identified metabolites are predictors of diabetic relapse.

**Animals:** Twenty cats in diabetic remission for a median of 101 days, and 22 healthy matched control cats.

**Methods:** Cats were admitted to a clinic, and casual blood glucose was recorded. After a 24 h fast, blood glucose concentration was measured, then a blood sample was taken for metabolomic (GCMS and LCMS) analyses. Three hours later, a simplified intravenous glucose tolerance test (1 g glucose/kg) was performed. Cats were monitored for diabetes relapse for at least 9 months (270 days) after baseline testing.

**Results:** Most cats in remission continued to display impaired glucose tolerance. Concentrations of 16 identified metabolites differed (*P* ≤ 0.05) between remission and control cats: 10 amino acids and stearic acid (all lower in remission cats), and glucose, glycine, xylitol, urea and carnitine (all higher in remission cats). Moderately close correlations were found between these 16 metabolites and variables assessing glycaemic responses (most |*r*| = 0.31 to 0.69). Five cats in remission relapsed during the study period. No metabolite was identified as a predictor of relapse.

**Conclusion and clinical importance:** This study shows that cats in diabetic remission have abnormal metabolism.

## Introduction

Diabetic remission, described previously by our group as maintaining euglycemia for at least two weeks after insulin therapy has ceased, can be achieved in many newly diagnosed feline diabetics, with highest remission rates reported in cats with type 2-like diabetes enrolled in studies with early intervention, intensive long-acting insulin therapy and low carbohydrate diets (1, 2). Approximately 25–30% of cats in remission will relapse and require further insulin therapy (1, 3, 4). In a previous study, the majority (76%) of diabetic cats in remission had impaired glucose tolerance (>5 h to return to ≤ 6.5 mmol/L), and some (19%) had impaired fasting glucose (≥ 6.6 mmol/L), indicating that these cats did not have normal glucose metabolism or clearance (4). Cut points for fasting blood glucose concentrations and normal glucose tolerance tests were determined based on data previously published from our laboratory (4, 5). Although validated cut points may eventually be useful in veterinary clinical practice to identify cats with altered glucose metabolism and at risk of developing diabetes, these concepts are largely confined to human medical practice. Except for the documentation of impaired glucose tolerance, the wider metabolic disturbances in diabetic cats in remission are poorly understood.

High-throughput metabolomics assays using mass spectrometry in combination with gas or liquid chromatography (GCMS and LCMS, respectively) enable measurement of a wide range of metabolites in body fluids, and provide a metabolite profile of the individual. Metabolomics analysis has been widely used to identify metabolite biomarkers of disease (6). Biomarkers that can predict the development of glucose intolerance and development of type 2 diabetes have been described in humans (7–9). For example, increased serum branched chain amino acids isoleucine, leucine and valine are associated with increased risk of development of diabetes (10).

The aims of this study were to compare the plasma concentration of metabolites between diabetic cats in remission and healthy control cats using GCMS and LCMS, and secondly to assess whether differentially expressed metabolites were predictors of diabetic relapse.

## Materials and Methods

### Study Design

This retrospective and prospective cohort study involved client-owned diabetic cats in remission presented to a feline-only veterinary hospital (“remission cats”), and clinically healthy owned cats presented to the feline-only or university hospital (“control cats”). Healthy control cats were frequency-matched with the diabetic cats based on age and body condition score. Previously insulin-treated diabetic cats in which insulin administration had ceased were retrospectively identified by reviewing practice records from January 2005 to April 2010, or were recruited prospectively from April 2010 to August 2012. Enrolment criteria were: 1. previously overtly diabetic cats that required insulin therapy; 2. at least one casual (non-fasted) glucose concentration ≤ 6.5 mmol/L (≤ 117 mg/dl) measured a minimum of two weeks after insulin administration was ceased; and 3 absence of clinical signs of diabetes prior to glucose tolerance testing as part of the current study. Remission date in this study was defined as 14 days after the date insulin administration ceased, and only cats in their first remission were included. At the time of initial diagnosis of diabetes, cats were only tested for other specific types of diabetes if they had relevant clinical signs. The cats in this cohort were assumed to have type 2 diabetes, unless tests showed otherwise. Other specific types of diabetes were not an exclusion criteria for this study. Remission dates for enrolled cats were from April 2006 to August 2012.

Clients filled out a questionnaire on admission that included type and quantity of foods fed to their cat. Calculations were made using information on dietary composition provided by the manufacturers to determine percentages on a dry matter basis of protein, carbohydrate, and fat estimated to be consumed by the cats.

After enrolment, all cats (remission and control) were admitted to the hospital and casual blood glucose recorded. Body weight and body condition score were recorded, an in-house urinalysis was performed, and a 3 ml jugular sample was collected and was sent to an external laboratory^1^ for haematology, serum biochemistry, serum fructosamine, total thyroxine (T4), and feline pancreasic lipase immunoreactivity.

Food was withheld for 24 h, and after overnight hospitalisation, another 3 ml blood sample was collected from the jugular vein for biochemical and metabolomic assay of plasma samples. Immediately after collection, blood was placed into an EDTA tube, centrifuged for 8 min at 1500 g and plasma separated into 1 ml Eppendorf tubes. Samples were stored at −80 degrees Celsius until they were transported on dry ice to the laboratory for analysis.

Additionally, cats had a simplified glucose tolerance test as previously described (4, 11). This was performed 3 h after blood collection for metabolomics and catheter placement to minimise the effects of stress hyperglycaemia (12). Briefly, the fasting blood glucose concentration was measured using a portable glucometer^2^ calibrated for cat blood from samples obtained from the ear (first preference), paw pad or jugular vein (depending on cat's temperament). Following measurement of fasting blood glucose, an intravenous bolus of glucose (50% glucose wt/vo) at 1 g/kg was administered over 1 min via a cephalic catheter placed 3 h before the test. Blood glucose was measured 2 h after glucose administration, and then hourly until blood glucose concentration was ≤ 6.5 mmol/L (≤ 117 mg/dL) or until 9 h after the test start, whichever occurred first. Time for glucose to return to baseline was set as time from test start until the first time that glucose concentration was measured as ≤ 6.5 mmol/L (4). Food was withheld for the duration of the glucose tolerance test.

### Metabolomic Analyses

Metabolic analyses and metabolite identification were performed by Metabolomics Australia (Melbourne, VIC, Australia).

### Gas Chromatography Mass Spectrometry (GCMS)

Plasma (50 μl) was transferred into a 1.5 ml Eppendorf® tube. Cold methanol (MeOH)(100 %, 4°C) (150 μl) and 1 μl of a quantitative internal standard (^13^C_6_-sorbitol/^13^${\text{C}}_{5}^{15}$N-Valine in water, 0.2 mg/ml) was added. The mixture was vortexed for 30 s and placed on ice for 10 min. Samples were centrifuged at 13,000 rpm for 10 min at room temperature (23°C) to precipitate protein. A (30 μl) aliquot was transferred into a glass insert and dried *in vacuo* for subsequent derivatisation.

Extracted plasma samples were re-dissolved in 10 μl of 30 mg/ml methoxyamine hydrochloride in pyridine and derivatised at 37°C for 120 min, mixing at 500 rpm. Samples were treated for 30 min with 20 μl *N, O*-*bis*-(trimethylsilyl)trifluoroacetamide (BSTFA) and 2 μl of a retention time standard mixture [0.029% (*v*/*v*) *n*-dodecane, *n*-pentadecane, *n*-nonadecane, *n*-docosane, *n*-octacosane, *n*-dotriacontane, *n*-hexatriacontane dissolved in pyridine], mixing at 500 rpm. Samples were rested for 60 min prior to injection.

Samples (1 μl) were injected using a hot needle technique into a GC-MS system comprised of a Gerstel 2.5.2 autosampler, a 7890A Agilent gas chromatograph and a 5975C Agilent quadrupole MS (Agilent, Santa Clara, USA).

GC was performed on a 30 m VF-5MS column with 0.2 μm film thickness and a 10 m Integra guard column (Varian, Inc, Victoria, Australia). Injection temperature was set to 250°C, MS transfer line 280°C, the ion source 250°C and the quadrupole 150°C. Helium carrier gas flow rate was set to 1 ml/min. The following temperature program was used: start at injection 35°C, hold for 2 min, then a 15°C/min oven temperature ramp to 325°C held for 3 min at 325°C. Data were evaluated using the Agilent MassHunter Workstation Software, Quantitative Analysis, Version B.05.00/Build 5.0.291.0 for GCMS. Individual metabolites were identified by matching mass spectra to the following libraries 1. Max-Planck-Institute for Plant Physiology library, Golm, Germany (http://csbdb.mpimp-golm.mpg.de/csbdb/dbma/msri.html) and the *in-house* Metabolomics Australia mass spectral library. All matching mass spectra were additionally verified by determination of the retention time with authentic standards. Relative response ratios (area of analyte divided by area of internal standard, ^13^C_6_-sorbitol) per volume of plasma were determined for each metabolite. If a specific metabolite had multiple trimethylsilyl (TMS) derivatives, the metabolite with the greater detector response and improved peak shape within the dynamic range of the instrument was selected.

### Liquid Chromatography Mass Spectrometry (LCMS)

Serum (50 μl) was diluted with acetonitrile (ACN)(150 μl, 0.1% formic acid) containing internal standards [^3^C_6_-sorbitol—(0.5 mg/ml), ^13^${\text{C}}_{5}^{15}$N-valine—(0.5 mg/ml), 2-aminoanthracene (0.25 mg/ml), pentafluorobenzoic acid (0.25 mg/ml)] then vortexed for 30 s and placed on ice for 10 min. The solution was centrifuged at 13,000 rpm for 5 min at 4°C then the supernatant transferred into a fresh vial prior to analysis.

An Agilent 6520A ESI-QqTOF-MS with attached Agilent 1200 series HPLC System comprised of Degasser, Pump, Auto-sampler with attached chiller cooled to 7°C. Mass spectra were collected in the positive (POS) and negative (NEG) ionisation modes across the mass range 70-1700 *m/z*, at a rate of 1.5–3 spectra/s. Instrument settings were as follows: gas temperature, 300°C; drying gas, 10 L/min; nebulizer, 40–45 psig; VCap, 3500 V; fragmentor; 150 V, Skinner, 65 V; OCT 1 RF Vpp, 750 V. Agilent Reference Mass solution was co-infused through dual ionisation source for online mass calibration.

For reverse phase (RP) chromatography an Agilent Zorbax Eclipse XDB-C18, 2.1 mm x 100 mm, 1.8 μm column was used with an injection volume of 5 μl. Solvent (A) 0.1% formic acid in Milli-Q water and solvent (B) 0.1% formic acid in can was used. A solvent flow rate of 0.4 ml/min with column temperature of 40°C and a 10 min linear gradient from 5% solvent (B) to 100% solvent (B) was used, followed by a 2 min hold at 100% solvent (B), then 5 min re-equilibration at 5% solvent (B) (total time of 17 min).

Mass features were extracted using Agilent Mass Hunter Profinder B.06.0 using a recursive feature extraction with the following settings: for positive ions +H, +Na and +K adducts, negative ions –H, +Cl adducts, with allowed neutral losses of H_2_O and H_3_PO_4_; an allowed charge state of 1-2 with a retention time window of 0.5 min and mass window of 15 ppm ±2 mDa. The data matrix was imported and features aligned using Agilent Mass Profiler Professional 13.1 (MPP) with the following settings for mass tolerance of 15 ppm ±2.0 mDa and retention time window of 0.7 min. The imported data was filtered by retaining entities that were found in at least 50% of any one condition. The aligned data matrix of neutral mass, retention time and response was exported and corrected for internal standard error manually prior to statistical analysis or metabolite identification.

Metabolites were identified in MPP using the ID Browser B.07.00 and were matched to the Metabolomics Australia in-house metabolite library using a mass match tolerance of 10 ppm mass error and retention time (RT) tolerance of 0.5 min against authentic standards. For positive ions +H, +Na and +K adducts; for negative ions –H, +Cl adducts were allowed, with possible neutral losses of H_2_O and H_3_PO_4_. A match was accepted when the overall weighted ID score was greater than 80 (maximum score = 100). Tentative identification of metabolites was undertaken by searching exported neutral mass features against the METLIN (https://metlin.scripps.edu) and LipidMaps (www.lipidmaps.org) online databases.

### Identification of Relapses in Remission Cats

After testing, remission cats were monitored for relapse using casual blood glucose measurements at home or in clinic. Relapse was defined as a blood glucose ≥11 mmol/L (198 mg/dl) on at least two occasions together with the presence of clinical signs consistent with diabetes (e.g., polyuria, polydipsia, polyphagia, and weight loss). Cats were monitored by observing blood glucose concentration (either by home monitoring or during health checks at the clinic) and clinical signs. The study endpoint was achieved when the cat relapsed, or, for non-relapsing cats, when it died from other causes or, for other cats, the most recent of the last recorded visit in clinic records (either the feline-only clinic or the practice that referred the cat to that clinic) or the last contact date with the owner.

### Statistical Analyses

Statistical analyses were performed using Stata (versions 13 and 15; StataCorp, College Station, Texas, USA). Principal component analyses were performed to visualise and compare the metabolomic data collectively between remission and control cats using Stata's -pca- command. Separate analyses were performed for GCMS, LCMS Reverse Phase Chromatography Negative Mode Ionisation (RP_NEG) and LCMS Reverse Phase Chromatorgraphy Positive Mode Ionisation (RP_POS) compounds using remission and control cats pooled. Concentrations were log-transformed for principal component analyses.

For each metabolite, concentrations were compared between remission and control cats using linear regression with cat age and body condition score (fitted as a continuous variables), breed (Burmese or non-Burmese) and sex (male or female; all cats were neutered), fitted as covariates. Concentrations were log-transformed before analyses. Coefficients for effects of remission were exponentiated (i.e., Euler's number was raised to the power of the coefficient), and the results interpreted as ratios of geometric means. These models were fitted using Stata's -regress- command. For metabolites where 15 or more of the 41 or 42 cats had the minimum reported concentration, instead of linear regression, proportions of cats that had concentrations above the minimum reported concentration were compared using exact logistic regression, fitted using Stata's -exlogistic- command. Cat breed, sex, age and body condition score were fitted as covariates as described above, and sufficient statistics were used. P-values were adjusted for multiple comparisons using the Benjamini-Hochberg step-up false-discovery rate method, implemented using the Etcetera module (version 3.02) in WinPepi (version 11.48) (14, 15).

To assess whether differences between remission and control cats were confounded by diet, for compounds that differed significantly between remission and control cats (*P*-value adjusted for multiple comparisons <0.05), point estimates were also calculated using the same models using cats that had dietary data. Additionally, separate models were used adjusting for either dietary protein % or dietary carbohydrate %. No adjustment for dietary fat % was performed as the means for this measure were similar in remission and control cats.

To compare remission and control cats independently of any effects of dietary carnitine, plasma carnitine concentrations were also compared between remission and control cats using the same model as described above excluding cats fed carnitine. Plasma carnitine concentrations were also compared between those cats fed and those not fed carnitine (*n* = 8 in each) within the remission cat group, again using the same approach as described above.

Correlations between each of the identified metabolites that differed significantly between remission and control cats and each of casual, fasting, and 2 h glucose, time for glucose to return to baseline, fPLI, and fructosamine were assessed using Pearson's correlation coefficients, or for correlations with time for glucose to return to baseline, Spearman's correlation coefficients. Fisher's transformation was used to calculate 95% confidence intervals. These were calculated using the -pwcorr-, -spearman- and -ci2- commands in Stata. *P*-values for correlation coefficients were adjusted for assessment of multiple pair-wise correlations in the same way as described above. For the 22 compounds with *p*-values <0.05 for differences between remission and control cats adjusted for multiple comparisons, concentrations were compared between relapsed and non-relapsed cats using linear regression as for comparisons between remission and control cats but with no covariates fitted. For each of these 22 compounds, less than 7 cats had minimal detectable concentrations.

## Results

Twenty cats in remission (median time in remission 110 days, range 10 days to 4 years) and 22 control cats were enrolled into the study (Table 1). Fourteen remission cats had presumed type 2 diabetes, four cats had a history of corticosteroid administration prior to developing clinic signs, one cat had corticosteroid administration coupled with severe pancreatitis on diagnosis and another cat had moderate pancreatitis at diagnosis. Diagnosis of pancreatitis was based on clinical signs and fPLI measured by an external laboratory. Glucose tolerance testing (GTT) was unable to be performed on one cat that had metabolomic testing and it was retained in the study for some analyses. Data for cat signalment, body condition score [scale of 1-9 (16)], glucose and fructosamine concentrations and feline pancreatic lipase immunoreactivity (FPLi) are summarised in Table 1. All control cats had fasting blood glucose <6.5 mmo/L (<117 mg/dl) and normal glucose tolerance as measured by blood glucose <6.5 mmol/L at 3 h (4). Of the diabetic cats in remission, 4/20 (20%) had impaired fasting glucose concentrations (fasting glucose 119–151 mg/dl vs. normal <117 mg/dl; 6.6–8.4 mmol/L vs. normal <6.5 mmol/L) and 14/19 (74%) had impaired glucose tolerance (Table 1). One remission cat did not reach the endpoint of glucose <6.5 mmol/L during its GTT. The lowest measured glucose concentration was 7.3 mmol/L (131 mg/dl) at 9 h after glucose administration, which was then determined to be its endpoint (4). In four of the six cats with suspected other specific types of diabetes, it was associated with corticosteroid administration without a concurrent diagnosis of pancreatitis, and three of these cats had impaired glucose tolerance and one had impaired fasting glucose concentrations. Of the two cats with pancreatitis at the time of initial diagnosis of diabetes, both (including the one with concurrent corticosteroid administration) had normal fasting glucose and glucose tolerance. Six of the 20 remission cats had International Renal Interest Society (IRIS) stage 2–3 chronic kidney disease. Of the 42 cats enrolled in the study (20 remission cats and 22 healthy control cats), GCMS metabolomic data were not available for 1 control cat and LCMS RP-NEG data were not available for 1 remission cat. In total, 978 compounds were statistically analyzed (the 61 compounds from GCMS that were identified by the laboratory reference library and all 917 identified and unidentified compounds from LCMS: 293 from RP_NEG and 624 from RP_POS).

**Table 1:** Signalment, body condition score, fasting and 2 h glucose concentrations during a GTT for cats in diabetic remission and control cats.

	**Remission (*n* = 20)**	**Control (*n* = 22)**
**SIGNALMENT**		
Sex	Male neutered 12; female spayed 8	Male neutered 12; female spayed 10
Median age at testing (range)	13 years (5 to 19 years)	10 years (5 to 18 years)
Median body condition score at testing (9 point scale) (range)	5.0 (4 to 7)	5.5 (4 to 8)
Breed	Domestic Short Hair 8, Burmese 8, Siamese 2, Australian Mist 1, Russian X 1	Domestic Short Hair 15, Burmese 4, Oriental 1, Abyssinian 1, Tonkinese 1
Median time in remission at testing (range)	101 days (10 days−4 years)^1^	Not applicable
% with normal fasting glucose concentration (≤ 6.5 mmol/L, 117 mg/dl)	80% (16/20)^2^	100% (22/22)
Mean fasting glucose concentration (range) (mmol/L)	5.8 (3.9 to 8.4)	4.4 (2.8 to 6.1)
Mean glucose concentration at 2 h (range) (mmol/L)	19.6 (7.6 to 25.9)^4^	8.6 (3.2 to 16.3)
% with normal glucose tolerance test^3^	26% (5/19)^4^	100% (22/22)
Mean fPLI^5^	5.8 (1 to 16)^6^	2.3 (0.5 to 5.1)^7^
Mean fructosamine^8^	267 (197 to 347)	250 (190 to 305)^7^

1 * Time from remission date (14 days after the date insulin administration ceased) to testing date*.

2 * The remaining four cats (20%) had fasting glucose concentrations of 6.6, 7.5, 7.8 and 8.4 mmol/L (119, 135, 140, and 151 mg/dl)*.

3 * Normal glucose tolerance test defined as glucose concentration ≤ 6.5 mmol/L (117 mg /dl) within three hours after 1 g/kg IV bolus of glucose administered*.

4 * The glucose tolerance test was not performed on one remission cat*.

5 * Laboratory reference range for fPLI was 0.1–3.5 ug/L within the normal range, 3.6–5.3 ug/L may indicate pancreatitis, and >5.4 ug/L consistent with pancreatitis*.

6 * fPLI results were not available for four remission cats*.

7 * fPLI and fructosamine results were not available for one control cat*.

8 * Laboratory reference range for fructosamine was 249–406 umol/L*.

**Figure 1:**
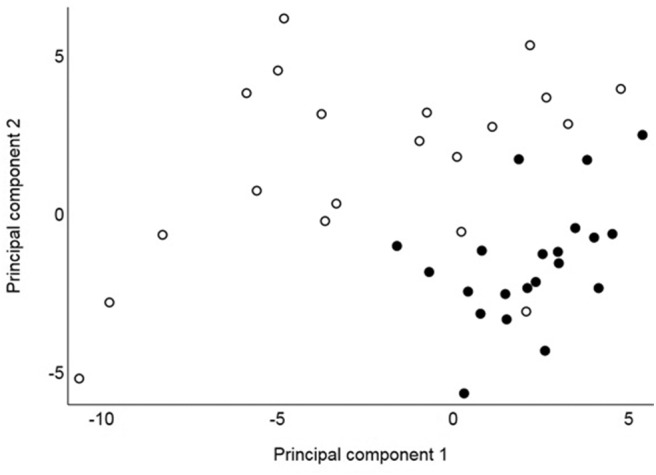
Principal component values from 41 cats for the first and second components based on concentrations of 61 identified GCMS compounds assessed in 20 cats in remission (hollow circles) and 21 healthy control cats (solid circles).

Principle component analysis revealed distinct clustering of collective GCMS metabolic phenotypes between remission and control cats with the first two components jointly highly discriminatory between these groups (Figure 1). The first two components accounted for 26% and 14%, respectively, of the total variance of 61. For the first component, 12 compounds had loadings above 0.2 (alanine, asparagine, glutamine, isoleucine, leucine, methionine, phenylalanine, proline, serine, threonine, tyrosine, and valine), and for the second component, eight compounds had loadings above 0.2 (gluconic acid, glycerol, glycerol-3-phosphate, phosphoric acid, threitol, threonic acid, 2,4-dihydroxybutanoic acid, and urea). The highest loadings for the first two components were 0.22 and 0.25, respectively, indicating that no single compound had a large influence on either component. Negative loadings were all close to zero (the most extreme was−0.14). These results show that when considered collectively, concentrations of GCMS compounds differ markedly between remission and control cats due to some of these 61 compounds. For each of RP_NEG and RP_POS LCMS compounds, collective metabolic phenotypes were not discriminatory between remission and control cats. After adjustment for multiple comparisons, serum concentrations of 22 compounds differed significantly (*P* <0.05) between remission and control cats, 16 of which were identified by the laboratory reference library (Table 2). The remaining six metabolites were not identified. Seventeen compounds (all RP_POS) were not compared between remission and control cats because concentrations had no or minimal variation between cats.

**Table 2:** Means and estimated ratios of means for metabolites significantly different between control and remission cats (*P* adjusted for multiple comparisons <0.05).

**Metabolite**	**Arithmetic mean (SD)**		**Geometric mean**		**Adjusted ratio of geometric means^1^ (95% CI)**	**P^2^**	**Adjusted ratio of geometric means^1^ for cats with dietary data (16 remission cats and 17 control cats)**		
	**Remission cats (*n* = 20)**	**Control cats (*n* = 22)**	**Remission cats (*n* = 20)**	**Control cats (*n* = 22)**			**Not adjusted for dietary variables**	**Also adjusted for dietary protein%**	**Also adjusted for dietary CHO%**
**GC-MS^3^ Amino Acids**									
Alanine	0.22 (0.21)	0.56 (0.29)	0.15	0.47	0.29 (0.17 to 0.51)	0.01	0.29 (0.16 to 0.52)	0.16 (0.05 to 0.53)	0.27 (0.11 to 0.67)
Asparagine	0.02 (0.03)	0.08 (0.03)	0.01	0.07	0.16 (0.09 to 0.27)	<0.01	0.14 (0.08 to 0.24)	0.13 (0.04 to 0.40)	0.15 (0.06 to 0.34)
Aspartate	0.03 (0.04)	0.10 (0.09)	0.01	0.06	0.08 (0.04 to 0.17)	<0.01	0.07 (0.03 to 0.16)	0.03 (0.01 to 0.18)	0.04 (0.01 to 0.15)
Glutamine	0.08 (0.14)	0.59 (0.28)	0.01	0.49	0.01 (0.00 to 0.05)	<0.01	0.11 (0.00 to 0.34)	0.00 (0.00 to 0.14)	0.01 (0.00 to 0.03)
Glycine	0.33 (0.14)	0.17 (0.10)	0.29	0.14	2.09 (1.39 to 3.14)	0.04	2.22 (1.40 to 3.52)	2.8 (1.11 to 7.06)	2.82 (1.41 to 5.64)
Leucine	0.74 (0.72)	1.31 (0.44)	0.35	1.18	0.22 (0.10 to 0.45)	0.01	0.20 (0.09 to 0.47)	0.14 (0.13 to 0.16)	0.19 (0.05 to 0.69)
Methionine	0.10 (0.10)	0.16 (0.08)	0.07	0.15	0.37 (0.23 to 0.60)	0.01	0.34 (0.20 to 0.57)	0.26 (0.09 to 0.74)	0.28 (0.13 to 0.62)
Proline	0.05 (0.05)	0.10 (0.05)	0.02	0.09	0.23 (0.10 to 0.50)	0.03	0.21 (0.09 to 0.46)	0.05 (0.01 to 0.22)	0.15 (0.04 to 0.49)
Serine	0.27 (0.26)	0.60 (0.22)	0.15	0.56	0.27 (0.14 to 0.50)	0.01	0.26 (0.13 to 0.50)	0.10 (0.03 to 0.35)	0.18 (0.07 to 0.47)
Tyrosine	0.06 (0.04)	0.12 (0.03)	0.04	0.12	0.26 (0.15 to 0.45)	<0.01	0.24 (0.13 to 0.45)	0.08 (0.02 to 0.24)	0.16 (0.06 to 0.40)
Valine	0.76 (0.70)	1.32 (0.53)	0.42	1.15	0.27 (0.14 to 0.51)	0.01	0.26 (0.12 to 0.53)	0.13 (0.03 to 0.56)	0.21 (0.07 to 0.62)
**GC-MS^3^ Fatty Acids**									
Octadecanoic acid	0.03 (0.02)	0.006 (0.02)	0.02	0.06	0.37 (0.27 to 0.50)	<0.01	0.35 (0.25 to 0.50)	0.30 (0.15 to 0.58)	0.37 (0.22 to 0.62)
**GC-MS^3^ Sugars and Sugar Alcohols**									
Glucose	44.14 (10.38)	33.8 (5.76)	42.93	33.32	1.30 (1.12 to 1.50)	0.05	1.28 (1.10 to 1.49)	1.21 (0.89 to 1.64)	1.29 (1.02 to 1.63)
Xylitol	0.01 (0.02)	0.03 (0.01)	0.01	0.02	0.19 (0.10 to 0.36)	<0.01	0.15 (0.08 to 0.26)	0.10 (0.03 to 0.31)	0.15 (0.06 to 0.35)
**GC-MS^3^ Other**									
Urea	4.39 (1.64)	2.66 (1.20)	4.10	2.45	1.70 (1.28 to 2.24)	0.02	1.77 (1.34 to 2.34)	2.02 (1.16 to 3.53)	1.85 (1.21 to 2.82)
**LC-MS^4^**									
Carnitine	0.033 (0.014)	0.015 (0.009)	0.030	0.013	2.50 (1.80 to 3.48)	<0.01	2.76 (1.96 to 3.87)	2.43 (1.22 to 4.81)	2.86 (1.70 to 4.80)

1 * Ratio of geometric mean for remission cats relative to that for control cats adjusted for cat age, breed (Burmese or non-Burmese), sex (male or female) and body condition score (95% CI not adjusted for multiple comparisons)*.

2 * P-value adjusted for multiple comparisons (P-value not adjusted for multiple comparisons were all <0.01)*.

3 * Gas chromatography mass spectrometry; data were not available for one control cat*.

4 * Liquid chromatography mass spectrometry*.

### Metabolites Detected With GCMS

**Figure 2:**
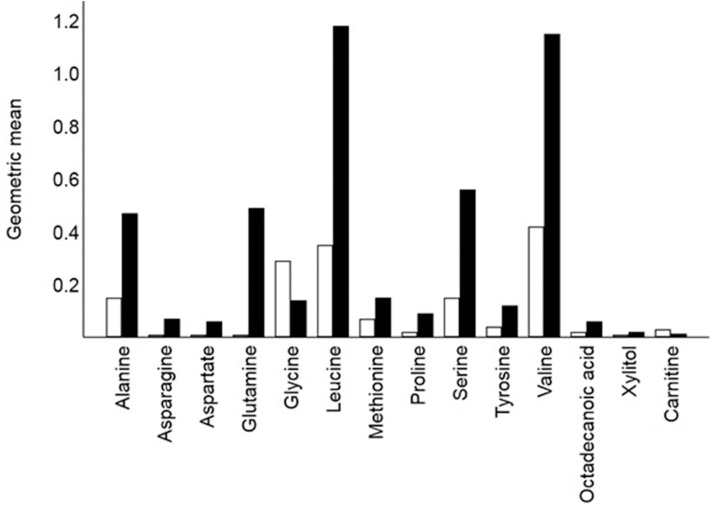
Crude geometric means for 14 of the 16 metabolites that differed signified between remission cats (hollow bars; *n* = 20) and control cats (solid bar *n* = 22). Glucose (means 42.9 and 33.3, respectively) and urea (means 4.1 and 2.5, respectively) also differed significantly between remission and control cats. Each bar shows the mean of the relative response ratios for each cat.

Fifteen identified GCMS metabolites differed significantly (*P* <0.05) between remission and control cats: 11 amino acids, 1 fatty acid (stearic acid), 1 sugar (glucose), 1 sugar alcohol (xylitol), and urea (Table 2, Figure 2).

#### Amino Acids and Derivatives

For most of the 11 amino acids (alanine, asparagine, aspartate, glutamine, methionine, proline, serine, tyrosine, and the branch-chain amino acids leucine, and valine), serum concentrations were significantly less amongst cats in remission, with means estimated to be more than 60% lower. In contrast, mean glycine concentration was estimated to be more than two-fold higher (Table 2). Most amino acid concentrations were negatively correlated (−0.3 < r < −0.7) with GTT results, either 2-h glucose (for 8 amino acids) or time for glucose to return to baseline (for 10 amino acids) (Table 3). Five amino acid concentrations were negatively correlated with either casual blood glucose and/or fasting blood glucose, and glycine was positively correlated with these measures (Table 3). Concentrations of other identified amino acids (glutamic acid, homoserine, isoleucine, lysine, phylalanine, pyroglutamic acid, threonine, and tryptophan) did not differ significantly between remission and control cats.

**Table 3:** Correlation coefficients (and *p*-values for testing null hypotheses that the correlation is 0) for correlations between selected metabolites and each of casual, fasting, and 2 h glucose, time for glucose to return to baseline, fPli and fructosamine.

**Metabolite**	**Plasma glucose concentrations**			**Time for glucose to return to baseline**	**fPLi**	**Fructosamine**
	**Casual**	**Fasting**	**2 h**			
**GC–MS Amino Acids**						
Alanine	−0.37	−0.26	−0.46	−0.57	0.01	−0.16
	*P* = 0.04	NS	*P* = 0.01	*P* <0.01	NS	NS
Asparagine	−0.34	−0.39	−0.55	−0.66	0.02	−0.23
	NS	*P* = 0.03	*P* <0.01	*P* <0.01	NS	NS
Aspartate	−0.34	−0.27	−0.35	−0.40	0.01	−0.22
	NS	NS	NS	*P* = 0.03	NS	NS
Glutamine	−0.44	−0.46	−0.59	−0.63	−0.15	−0.37
	*P* = 0.02	*P* = 0.01	*P* <0.01	*P* <0.01	NS	*P* = 0.04
Glycine	0.39	0.48	0.35	0.34	0.33	0.55
	*P* = 0.03	*P* = 0.01	NS	NS	NS	*P* <0.01
Leucine	−0.37	−0.39	−0.43	−0.49	0.22	−0.23
	*P* = 0.04	*P* = 0.03	*P* = 0.02	*P* = 0.01	NS	NS
Methionine	−0.22	−0.21	−0.28	−0.41	0.33	−0.25
	NS	NS	NS	*P* = 0.03	NS	NS
Proline	−0.33	−0.28	−0.44	−0.51	0.15	−0.17
	NS	NS	*P* = 0.02	*P* = 0.01	NS	NS
Serine	−0.33	−0.22	−0.46	−0.52	0.06	−0.14
	NS	NS	*P* 0.01	*P* <0.01	NS	NS
Tyrosine	−0.47	−0.44	−0.52	−0.58	0.05	−0.26
	*P* = 0.01	*P* = 0.01	*P* <0.01	*P* <0.01	NS	NS
Valine	−0.39	−0.43	−0.40	−0.46	0.25	−0.34
	*P* = 0.03	*P* = 0.02	*P* = 0.03	*P* = 0.01	NS	NS
**GC–MS Fatty Acids and Sterols**						
OctadecanoicAcid	−0.52	−0.50	−0.69	−0.67	−0.31	−0.11
	*P* <0.01	*P* <0.01	*P* <0.01	*P* <0.01	NS	NS
**GC–MS Sugars and Sugar Alcohols**						
Glucose	0.29	0.41	0.49	0.55	0.36	0.19
	NS	*P* = 0.02	*P* = 0.01	*P* <0.01	NS	NS
Xylitol	−0.34	−0.46	−0.46	−0.40	0.19	−0.34
	NS	*P* = 0.02	*P* = 0.01	*P* = 0.03	NS	NS
**GC–MS Other Metabolites**						
Urea	0.24	0.34	0.43	0.42	0.08	0.05
	NS	NS	*P* = 0.02	*P* = 0.02	NS	NS
**LC–MS Metabolites**						
Carnitine	0.25	0.31	0.46	0.42	0.15	0.14
	NS	NS	*P* = 0.01	*P* = 0.02	NS	NS

*Not significant (NS) indicates P > 0.05*.

#### Fatty Acids and Sterols

The mean concentration of one fatty acid, octadecanoic (stearic) acid, was decreased by an estimated 63% in cats in remission (Table 2), and was negatively correlated (−0.5 < r < −0.7) with all glucose variables (Table 3). Concentrations of other identified fatty acids and sterols (1-campesterol, 1-monohexadecanoylglycerol, 1-monooctadecanoylglycerol, 2-monooleoglycerol, 5-alpha-chloestan-3-ol, beta-sitosterol, cholesterol, hexadecanoic acid, hexadecenoic acid, octadecadienoic acid, octadecenoic acid, and tetradecanoate) did not differ significantly between remission and control cats.

#### Sugars and Sugar Alcohols

The mean concentration of glucose measured by GCMS was an estimated 30% higher (*P* <0.05) in remission cats (Table 2), and as expected correlated with fasting glucose measured 3 h after the metabolomics sample was taken using a hand-held glucometer, and 2 h glucose and time for glucose to return to baseline during GTT (Table 3). The mean concentration of the sugar alcohol, xylitol, was an estimated 81% lower (*P* <0.01) in remission cats (Table 2), and was negatively correlated (−0.4 < r < −0.5) with all glucose variables, except casual blood glucose (Table 3). Concentrations of other identified sugars (fructose, galactinol, inositol, maltose, and threitol) did not differ significantly between remission and control cats.

#### Organic Acids

Several organic acids were identified (2,4-dihydroxybutanoic acid, 2-amino-butyric acid, 2-ketoglutaric acid, aconitic acid, citric acid, fumaric acid, gluconic acid, glyceric acid, lactic acid, malic acid, picolinic acid, pyruvic acid, succinic acid, and threonic acid), but concentrations of these did not differ significantly between remission and control cats.

#### Other Compounds

The mean concentration of urea was an estimated 70% higher (*P* <0.05) in remission cats (Table 2). In further analysis, urea remained significantly (*P* <0.05) higher in remission cats even after exclusion of six remission cats with IRIS stage > 2 chronic kidney disease (creatinine ≥140 μmol/L and USG ≤ 1.035)^3^ Urea was positively correlated (*r* = 0.4) with both 2 h glucose and time for glucose to return to baseline during GTT (Table 3). Concentrations of other identified compounds (glycerol, glycerol-3-phosphate, phosphoric acid, phosphoric acid monomethylester, pyrophosphate, serotonin, and tocopherol) did not differ significantly between remission and control cats.

### Correlations Between Metabolites and fPLi and Fructosamine

Only two identified GCMS metabolites were significantly correlated with fructosamine (Table 3). The amino acid, glutamine, had a negative correlation (*r* = −0.4), whilst glycine was positively correlated (*r* = 0.6) with fructosamine. There were no significant correlations between any identified GCMS metabolite and fPLI.

### Metabolites Detected With Lcms

Mean concentrations for seven metabolites differed significantly between remission and control cats when analysed using LCMS, although only one (the amino-acid derivative, carnitine) was identified. The mean concentration of carnitine was estimated to be 250% higher (*P* <0.01) in remission cats (Table 2, Figure 2), and was positively correlated with each of 2 h glucose and time for glucose to return to baseline during GTT (*r* = 0.46 and 0.42, respectively; Table 3).

#### Associations With Relapse Amongst Remission Cats

Of the 20 remission cats enrolled in the study, 19 were monitored for relapse; 5 of those relapsed 50, 58, 103, 230, and 257 days after testing. Of the remaining 14 cats, 13 were monitored for between 270 and 1133 days (median 614 days), and 1 cat died at day 253. None of the metabolites that differed significantly between remission and control cats was a significant (*P* <0.05) predictor of relapse.

#### Effects of Diet

Dietary information was available for 16/20 (80%) remission cats and 17/22 (77%) control cats. Mean dietary protein content was higher (50 vs. 31%) and mean carbohydrate content lower (25 vs. 40%) in remission cats and control cats, respectively, whilst mean fat contents were similar (17 vs. 18%). Adjustment for either dietary protein or dietary carbohydrate did not change whether identified metabolites were increased or decreased, but in some instances the difference between remission and control cats was more pronounced after adjustment for protein intake (Table 2). Carnitine was present in the diet of half of the remission cats, but no control cats. However, the increase in mean serum carnitine concentration in remission cats compared to controls was similar amongst cats not fed carnitine (ratio of geometric means 3.11; 95% CI 1.85 to 5.22) to when all cats were included (Table 2).

## Discussion

A metabolomics (GCMS and LCMS) approach was used to identify novel metabolites of diabetic cats in remission. The plasma concentrations of many glucogenic amino acids were markedly reduced in remission cats, except glycine which was increased. Urea and carnitine were also increased, and octadecanoic (stearic) acid was decreased. Associations were found between identified metabolites and glycaemic measures in this cohort of cats in remission, of which 74% continued to display impaired glucose tolerance and 20% had mildly impaired fasting glucose. None of the metabolites proved to be a predictor of relapse, although they provide new insights into altered metabolism in diabetes in cats.

In the current study, mean plasma glucose concentrations assessed by GCMS analysis were an estimated 30% higher in cats in diabetic remission compared to control cats, confirming blood glucose measurements 3 h later using a handheld glucometer (4). This increase in fasting blood glucose concentration in remission cats is most likely due to increased hepatic gluconeogenesis as a result of unresolved insulin resistance (17). On average, diabetic cats are six times less sensitive to insulin compared to normal cats (17). Hepatic gluconeogenesis, which is normally inhibited by insulin, is increased in insulin resistance in cats (18). Based on the findings of this current study, cats in diabetic remission have a decrease in many glucogenic amino acids (alanine, asparagine, aspartate, glutamine, methionine, proline, serine, tyrosine, and valine), consistent with the hypothesis of increased consumption for gluconeogenesis in these animals. Indeed, all of these amino acids were negatively associated with casual and fasting blood glucose, 2 h blood glucose and return to baseline during a GTT. Alternatively, the decrease in glucogenic amino acids observed in cats in remission might simply reflect a shift towards amino acid metabolism due to decreased carbohydrate metabolism. Lower amino acid concentrations may reflect increased oxidation of amino acids as substrates in the tricarboxylic acid (TCA) cycle, on a background of reduced glycolysis, as suggested in human diabetics (19). Reduced concentrations of glucogenic precursors alanine, glutamine, serine, glycine and arginine have been previously reported in diabetic humans and mice (19, 20). Altogether, the reduction in many glucogenic amino acids in cats in diabetic remission in the current study supports the idea of continued dysregulation of glucose and amino acid metabolism.

An unexpected result in the current study was glycine, which was increased an estimated 2-fold in remission cats. Glycine levels are decreased in humans with type 2 diabetes or impaired glucose tolerance, with reduced glycine concentrations being considered a risk factor for the development of type 2 diabetes in humans (8, 9, 21, 22). Low glycine has been correlated with increased 2-h blood glucose concentration following a GTT in humans, and with increased fasting and 2-h glucose concentrations in rats (9, 23) Also in cats, glycine is known to be an efficient gluconeogenic precursor, even better than in rats (24). Therefore, increased glycine in cats in remission suggest peculiar glycine metabolism, perhaps unique to diabetic remission. A possible explanation is increased glyoxylate metabolism, with glycine being formed from glyoxylate via transamination utilising alanine as a donor. In this regard, we have noted abnormal glycolate-glyoxylate metabolism, together with dysregulated glucose metabolism, in both senior obese and Burmese cats at high risk of developing diabetes, although in both these cat groups, plasma glycine was normal (5). In humans, increased dietary glycine intake has beneficial effects in obesity and type 2 diabetes, including improving insulin sensitivity (25, 26). However, here in cats in diabetic remission, glycine concentrations were positively correlated with both casual and fasting glucose. Therefore, the functional significance of increased glycine in remission cats is unclear.

Leucine and valine, two branched-chain amino acids (BCAAs), were significantly decreased in remission cats. In humans and diabetic mice, increases in BCAAs (including isoleucine) are associated with impaired fasting glucose and type 2 diabetes (10, 20, 27). BCAAs are thought to modulate insulin secretion, and increased circulating BCAAs may promote insulin resistance by disrupting insulin signalling in skeletal muscle (10). Remission cats had decreased leucine and valine levels in spite of impaired fasting glucose and delayed glucose tolerance. Obesity has been linked to increased BCAAs in humans(22), and mean body condition score of our remission cats was 5 (out of 9, range 5–7) and control cats was 5.5 (range 4–8) (23). However, body condition was adjusted for in the comparison of remission and control cats, so is unlikely to explain why leucine and valine were reduced in remission cats.

High levels of saturated fatty acids are a risk factor for diabetes in humans, and high dietary intake is associated with obesity and increased risk of developing diabetes (28, 29). Octadecanoic (stearic) acid, a saturated fatty acid, was the only lipid significantly different between groups, being reduced in remission cats and having negative correlations with glucose parameters. This fatty acid was also decreased in Burmese cats at higher risk of developing diabetes, and thus reductions in stearic acid levels might reflect quite different lipid metabolism in diabetic cats (5). However, feeding cats a stearic rich, high fat diet increases hepatic lipid accumulation and other early signs of liver damage (30). More work is required to investigate lipid metabolism in obesity and diabetes in cats.

Urea was also increased in remission cats. Although a subset of remission cats did have renal disease (thus decreased excretion of urea), the increase in plasma urea was still significant after analysis without these cats. In part, the increase in urea might be associated with an increase in alanine metabolism through the glucose-alanine cycle, which converts pyruvate in muscle to alanine, which is then transported to the liver, where it is then converted back to pyruvate and used as a substrate for gluconeogenesis or a precursor for the TCA cycle (31). A by-product of this cycle is urea, produced in the liver. In men, urea was one of many markers found to be a discriminator of impaired fasting glucose, and similarly in the cats in remission, plasma urea showed a positive relationship with glucose results in GTT (32).

Mean concentration of xylitol was decreased by an estimated 80% in remission cats, and xylitol was negatively correlated to glucose variables. Diabetic rats fed a 10% xylitol solution exhibit improved glucose tolerance and pancreatic islet morphology (33, 34). Xylitol ingestion in dogs is potentially lethal, stimulating insulin secretion, and causing marked hypoglycaemia, although this has never been reported in cats (35). Further research is needed to determine if impaired glucose tolerance results in decreased xylitol, or if decreased xylitol results in impaired glucose tolerance.

The mean concentration of the amino-acid derivative, carnitine, was an estimated 250% higher in remission cats, and was also positively correlated with glucose measures in GTT. Carnitine is absorbed through dietary intake or is synthesised in the liver, and is an essential cofactor of fatty acid metabolism, assisting in the transport of acetyl Co-A out of mitochondria. Accumulation of acetyl Co-A has been implicated in insulin resistance in skeletal and cardiac muscle of humans and rodents (36, 37). In human and rodent studies, supplementation with carnitine improved glucose parameters in diabetic subjects regardless of the route given (IV or oral) (36). Whilst 50% of remission cats ate a diet with carnitine added, the increase in serum carnitine in remission cats was found to be independent of dietary carnitine.

Diet composition, in particular high protein content, has been shown to alter metabolic profiles in healthy cats fed a specific diet twice a day for 15 days with samples collected after an overnight fast on the 16th day (38). However, the post-prandial period in cats can be up to 24 h, and therefore, following an overnight fast, cats were likely still in the post-prandial period, which may account for the dietary differences seen in that study (39). All cats in our study were fasted 24 h prior to testing, which would be expected to remove any post-prandial effects of diet. Most human studies reporting metabolomics data do not limit patients to a specific diet providing adequate fasting is observed, and veterinary studies using other modalities (even clinical biochemistry) to measure metabolites in diseases such as diabetes or obesity, typically either do not have all patients on the same diet (8, 9, 27). Obviously, where there might be a postprandial effect, either overnight or 24 h fasting is accepted as sufficient to minimise the dietary effect.

Whilst diabetic diets (high in protein and lower in carbohydrate) were recommended to owners of cats in diabetic remission (excepting cases with renal disease, where a renal diet was advised), there was no provision in this study to supply specific food, and thus diet was left up to owner discretion. Although the remission group in the current study did have higher mean protein content in their diet (50 vs. 31% in the control cats), the metabolite results when adjusted for diet showed either similar or even greater differences in metabolites compared to remission cats. Requiring cats to have eaten a specific diet would limit the suitability of the metabolites discovered for future use as diagnostic measures in veterinary clinics.

This study did not exclude cats based on the type of diabetes present. Our aim was to examine diabetic cats in remission, rather than to confine the cohort to type 2 diabetics, because of the difficulty in being confident of this diagnosis. Whilst the majority (72%) of cats were presumed to have type 2 diabetes, screening for other specific types of diabetes such as acromegaly was not routinely performed. Although five cats had therapeutic doses of glucocorticoids, these do not normally induce diabetes in cats, and those with steroid-induced diabetes likely have underlying β-cell dysfunction, which may be associated with the same process causing type 2 diabetes. In humans diabetics, pre-existing β-cell dysfunction causing defects in insulin secretion are normally present when corticosteroid administration induces diabetes (40). However, corticosteroid-induced diabetes is classified as an “other specific type of diabetes” by the American Diabetes Association, as it is possible that the underlying pathophysiologic process is easier to reverse (41). Significantly higher remission rates are reported in cats that had corticosteroids administered in the 6 months prior to diagnosis (1). It has been suggested that these cats should be classified as “other specific type” of diabetes, but if they relapse following remission they are classified as type 2. Of the four cats with previous corticosteroid administration and without concurrent pancreatitis, only one relapsed following additional corticosteroid administration for palliation of a spinal lesion. The major limitation of this study was small sample size, which may have limited the ability to detect some variables associated with relapse, if any associations exist. Metabolomic analysis did not take into account the type of diabetes prior to remission due to small numbers. A larger scale study would allow for comparison of identified metabolites between types of diabetes and control cats. Finally, limitations on the total number of available authentic standards limited the total number of metabolites able to be positively identified by LCMS. This study focused on differences between cats in diabetic remission and healthy control cats. Whilst outside the scope of this study, a comparison between diabetic cats and diabetic cats in remission would add further understanding to this field.

In conclusion, this study shows that diabetic cats in remission have major differences in broader metabolism, compared to control cats. An improved understanding of abnormal metabolism will enable future testing of markers related to abnormal glucose metabolism and/or allow for targeted therapies to be developed to improve glucose tolerance in these cats.

## Data Availability Statement

All datasets generated for this study are available on request.

## Ethics Statement

The animal study was reviewed and approved by The University of Queensland Animal Ethics Animal Welfare Unit, Research and Research Training Division. Written informed consent was obtained from the owners for the participation of their animals in this study.

## Author Contributions

SG: conception and design, analysis and interpretation of data and drafting the article for important intellectual content. This study forms part of SG's Ph.D. research. ZR, JM, SA, and BB: analysis and interpretation of data and critically revising the article for important intellectual content. UR and DD: analysis and interpretation of data. JR: conception and design, interpretation of data, critically revising the article for important intellectual content and final approval of the version to be published.

## Conflict of Interest

JM was employed by the company Jemora Pty Ltd. ZR was employed by the company Nestlé Purina, and is currently employed by Sadara Chemical Company. The authors declare that this study received funding from Nestlé Purina. The funder provided advice on interpretation of the data and assistance with reviewing the article for intellectual content, but was not involved in the study design, data collection, or the decision to submit it for publication.

## Footnotes

^1^IDEXX Laboratories, Brisbane, QLD, Australia

^2^AlphaTRAK, Abbott Animal Health (13).

^3^International Renal Interest Society: http://www.iris-kidney.com
